# Acid-Induced Inflammatory Cytokines in Osteoblasts: A Guided Path to Osteolysis in Bone Metastasis

**DOI:** 10.3389/fcell.2021.678532

**Published:** 2021-05-28

**Authors:** Gemma Di Pompo, Costantino Errani, Robert Gillies, Laura Mercatali, Toni Ibrahim, Jacopo Tamanti, Nicola Baldini, Sofia Avnet

**Affiliations:** ^1^Biomedical Science and Technologies Lab, IRCCS Istituto Ortopedico Rizzoli, Bologna, Italy; ^2^Orthopaedic Oncology Surgical Unit, IRCCS Istituto Ortopedico Rizzoli, Bologna, Italy; ^3^Department of Cancer Physiology, H. Lee Moffitt Cancer Center and Research Institute, Tampa, FL, United States; ^4^Osteoncology and Rare Tumors Center, IRCCS Istituto Romagnolo Per Lo Studio Dei Tumori (IRST) “Dino Amadori”, Meldola, Italy; ^5^National Tumor Assistance (ANT) Foundation, Bologna, Italy; ^6^Department of Biomedical and Neuromotor Sciences, University of Bologna, Bologna, Italy

**Keywords:** bone metastasis, intratumoral acidosis, osteoclasts, osteoblasts, interleukin 8, interleukin 6, tocilizumab, cancer microenvironment

## Abstract

Bone metastasis (BM) is a dismal complication of cancer that frequently occurs in patients with advanced carcinomas and that often manifests as an osteolytic lesion. In bone, tumor cells promote an imbalance in bone remodeling via the release of growth factors that, directly or indirectly, stimulate osteoclast resorption activity. However, carcinoma cells are also characterized by an altered metabolism responsible for a decrease of extracellular pH, which, in turn, directly intensifies osteoclast bone erosion. Here, we speculated that tumor-derived acidosis causes the osteoblast–osteoclast uncoupling in BM by modulating the pro-osteoclastogenic phenotype of osteoblasts. According to our results, a low pH recruits osteoclast precursors and promotes their differentiation through the secretome of acid-stressed osteoblasts that includes pro-osteoclastogenic factors and inflammatory mediators, such as RANKL, M-CSF, TNF, IL-6, and, above the others, IL-8. The treatment with the anti-IL-6R antibody tocilizumab or with an anti-IL-8 antibody reverted this effect. Finally, in a series of BM patients, circulating levels of the osteolytic marker TRACP5b significantly correlated with IL-8. Our findings brought out that tumor-derived acidosis promotes excessive osteolysis at least in part by inducing an inflammatory phenotype in osteoblasts, and these results strengthen the use of anti-IL-6 or anti-IL-8 strategies to treat osteolysis in BM.

## Introduction

Up to 70% of advanced patients with prostate and breast carcinomas develop bone metastasis (BM) at the later stages of the diseases (Roodman, 2004). Thyroid, lung, and kidney carcinomas also have a significant osteotropism for secondaries (Coleman, 2001; Avnet et al., 2007). Unfortunately, once carcinoma spreads to the skeleton, it causes devastating clinical complications and skeletal-related events, including pain, fractures, spinal cord compression, and hypercalcemia (Weilbaecher et al., 2011; Di Pompo et al., 2017).

Circulating carcinoma cells are attracted to colonize bone by favorable conditions. It is widely accepted that BM formation is induced by a vicious cycle: tumor-secreted soluble factors disrupt the regular cycle of bone remodeling by promoting an imbalance between bone-forming osteoblasts (OB) and bone-resorbing osteoclasts (OC), thereby allowing the invasion of cancer cells (Kingsley et al., 2007). Although osteogenic lesions may also occur, osteolytic metastases are more frequent. In this case, tumor cells directly or indirectly induce the release of OC-activating factors through several mechanisms that have been deeply investigated (Yin et al., 2005). Among these are, *inter alia*, receptor activator of nuclear factor kappa-B ligand (RANKL) that is the most relevant pro-osteoclastogenic paracrine factor, transforming growth factor-beta (TGF-β), vascular endothelial growth factor (VEGF), insulin-like growth factors (IGFs), parathyroid-related protein (PTHrP), interleukin 11 (IL11), tumor-necrosis factor-alpha (TNF-α), prostaglandin E2 (PGE2), runt-related transcription factor 2 (Runx2), and Wnt pathway components (Kingsley et al., 2007). Furthermore, we recently demonstrated that tumor-derived metabolites may also regulate the activity of bone cells as lactate released by the glycolytic breast carcinoma cells fuels the metabolic activity of bone-resorbing OC (Lemma et al., 2017).

Tumor-induced pro-osteoclastogenic pathways are the leading focus of investigation for BM and the main target for the most innovative therapeutic interventions. In the clinical setting, bisphosphonate-based anti-resorptive drugs and a humanized anti-RANKL antibody (Denosumab) are the most used (von Moos et al., 2019). However, these treatments are incompletely effective, and thus, more detailed comprehension of the pathogenesis of BM would greatly expedite the identification of alternative approaches.

As for other types of cancers, the development and progression of BM are strongly affected by biophysical factors that are crucial for the formation of the metastatic niche, including hypoxia (Kingsley et al., 2007; Johnson et al., 2017). Hypoxia may derive from the altered growth and oxygen consumption rate of cancer cells and, specifically for bone cancers, by distinct features of the bone microenvironment. Indeed, hypoxia is a relevant contributor to bone biology and physiology (Arnett, 2010). In the medullary cavity of animal models, pO_2_ values range from 11.7 to 31.7 mmHg (1.5–4.2%), with a mean of 20.4 mmHg (2.7%) (Spencer et al., 2014). Acidosis is a further physicochemical factor that characterizes BM microenvironment. It is due to (1) the metabolic switch to glycolytic metabolism, induced by hypoxia or by the Warburg effect that, in turn, causes the accumulation of lactic acid and protons in the extracellular space (Yoneda et al., 2015; Corbet and Feron, 2017; Kolosenko et al., 2017; Pillai et al., 2019), (2) the hydration of excessive CO_2_ in the more oxidative areas of the tumor (Corbet and Feron, 2017), and (3) the active release of protons by OC via the plasma membrane (a3 isoform) vacuolar-H^+^-ATPase (V-ATPase) to resorb bone (Yoneda et al., 2015). Although tumor acidosis in BM is already widely accepted (Nagae et al., 2007; Nishisho et al., 2011; Yoneda et al., 2011, 2015; Huang et al., 2016; Sharma et al., 2016; Hiasa et al., 2017; Ji et al., 2019), the average intratumoral pH has not been assessed so far due to technical limitations that cannot be overcome. In fact, intratumoral pH should be directly measured by inserting microelectrodes during surgery, a non-canonical procedure (Engin et al., 1995), or, indirectly, by non-invasive imaging techniques such as the MRI-CEST (Longo et al., 2016), which is not yet approved for clinical use. Intratumoral acidity was the rule in other types of cancer, including adenocarcinoma (pHe 6.93 ± 0.08, range 5.66–7.78), soft tissue sarcoma (pH 7.01 ± 0.21, 6.25–7.45), squamous cell carcinoma (pH 7.16 ± 0.08, 6.2–7.6), and malignant melanoma (pH 7.36 ± 0.1, 6.98–7.77) (Engin et al., 1995). In BM, the direct measurement might be performed *ex vivo*, after surgical removal, but not following conservative procedures such as palliative stabilization of impending fractures, a common event in BM treatment. Regarding MRI imaging, it is possible to speculate that extracellular pH resulting from the altered metabolism of BM deriving from breast cancer may well correspond to that of primary lesions (Longo et al., 2016).

In broad terms, tumor-derived acidosis is a hallmark of cancer that modulates tumor stemness, invasion, invadopodia formation, metastasis, anti-cancer immune response, and response to therapy (Cardone et al., 2005; Avnet et al., 2017; Kolosenko et al., 2017; Damgaci et al., 2018). Similarly, for tumors that form and develop in the skeletal tissue, microenvironmental acidosis is responsible for the promotion of stemness (Avnet et al., 2017), survival under hostile conditions (Avnet et al., 2019a), drug resistance, and a more invasive tumor phenotype (Cardone et al., 2005; Avnet et al., 2013, 2016, 2017). Notably, in bone, local variations of extracellular pH are tightly monitored and adjusted on a regular base since they may affect both OB and OC differentiation and activity. In more detail, extracellular acidification increases OC activity and the formation of resorption pits by OC [maximal stimulus at pH < 6.9 (Arnett and Dempster, 1986; Granchi et al., 2017)]. Furthermore, acidosis upregulates the activity of cathepsin K (required for organic matrix degradation), tartrate-resistant acid phosphatase (TRACP), and TNF receptor-associated factor 6 (TRAF6) (Shibutani and Heersche, 1993; Arnett, 2010; Yuan et al., 2016). Thus, in the context of BM, low extracellular pH may be an essential requirement for the initiation of the osteolytic process [for a more detailed update on the current state-of-the-art on acid-related effects in BM, see our recent review (Avnet et al., 2019b)]. *Vice versa*, osteogenesis (OB differentiation, mineralization, and bone formation) is affected by a low extracellular pH (Brandao-Burch et al., 2005; Massa et al., 2017). Altogether, these observations warrant that the role of local acidosis in bone physiology and BM pathology has been mostly defined. Per contra, the effect of acidosis on OB–OC paracrine activity has been less acquainted. On this regard, it is important to note that a decrease of local pH is *per se* an inflammatory stimulus that causes the release of various enzymes during phagocytosis, the damage of vasculature and other surrounding tissues, and the prolonging of the healing process by stimulating new inflammatory reactions (Riemann et al., 2016). In turn, when stimulated, OB has been recently suggested to contribute to the persistence of the inflammatory response and behave like inflammatory cells through the synthesis and the release of pro-inflammatory cytokines, like interleukin 8 (IL-8), the monocyte chemoattractant protein-1 (MCP-1), or interleukin 6 (IL-6) (Marriott et al., 2004; Dapunt et al., 2016) that, in turn, may exacerbate the pro-osteolytic cascade.

In this study, we speculated that tumor-derived acidosis causes the OB–OC uncoupling in BM by directly inducing a pro-osteoclastogenic phenotype in osteoblasts. Following this hypothesis, we (1) corroborated the data regarding the acidifying activity of carcinoma cells prone to metastasize to bone. We demonstrated that (2) acidosis directly promotes the recruitment of OC precursors, and (3) the release of OB-derived inflammatory secretome that, in turn, (4) modulates the progression of osteolysis. Finally, by analyzing serum samples from metastatic patients, (5) we also correlated circulating biomarkers of osteolysis with the circulating levels of those inflammatory cytokines that may be released at the site of BM after the acidic stimulus.

## Materials and Methods

### Cell Cultures

Tumor cells: Human breast carcinoma (MCF7, and MDA-MB-231) and renal carcinoma (Caki-1, and ACHN) cell lines were purchased from the American Type Cell Culture Collection (ATCC). CRBM-1990 cells were previously isolated and characterized from a BM of renal cell carcinoma (Avnet et al., 2004). Breast and renal carcinoma cell lines were maintained in complete Iscove’s modified Dulbecco’s medium (IMDM). Complete medium was supplemented with 10% heat-inactivated fetal bovine serum (FBS, Euroclone), plus 100 U/ml of penicillin, and 0.1 mg/ml of streptomycin (Life Technologies). Cells were cultured at 37°C, in a humidified atmosphere of 5% CO_2_. Unless otherwise indicated, cells were maintained at pH 7.4.

Human osteoblasts: hOB from healthy donors were purchased from VWR International PBI. Human MSCs from bone marrow (BM-MSCs) were purchased from Lonza (Euroclone). hOBs were maintained in complete Dulbecco’s modified Eagle’s medium (DMEM), and BM-MSC in complete minimum essential medium Eagle’s alpha modified (α-MEM). Complete media were supplemented with 10% heat-inactivated fetal bovine serum (FBS, Euroclone), plus 100 U/ml of penicillin, and 0.1 mg/ml of streptomycin (Life Technologies). Cells were cultured at 37°C, in a humidified atmosphere of 5% CO_2_. Unless otherwise indicated, cells were maintained at pH 7.4.

Human osteoclasts: Human OC (hOC) were derived from buffy-coats of at least two healthy donors (AVIS, Bologna), as previously described (Avnet et al., 2007). Peripheral blood mononuclear cells (PBMC) were isolated on Ficoll-Hystopaque gradient (GE Healthcare) and washed with PBS. To obtain differentiated hOC, isolated cells were resuspended in complete high-glucose DMEM (Euroclone) supplemented with 10% heat-inactivated characterized FBS (Celbio), plus 100 U/ml of penicillin, and 0.1 mg/ml of streptomycin (Life Technologies) (OC complete medium), and plated on different supports, depending on the type of assay, at a cell density of 3 × 10^6^ cells/cm^2^. PBMCs were incubated at 37°C in a humidified atmosphere of 5% CO_2_. After 2 h, non-adherent cells were removed, and the medium was replaced with different media depending on the type of assay.

Human CD14^+^ monocytes: To obtain an enriched population of OC precursors, CD14^+^ cells were derived from PBMCs isolated from buffy-coats of two healthy donors by immunomagnetic separation using an anti-CD14 monoclonal antibody (MiniMACS; Myltenyi Biotec). Briefly, PBMCs were isolated from buffy-coats, as described above, washed with MACS buffer (PBS at pH 7.2, supplemented with 0.5% bovine serum albumin and 2 mmol/L ethylene diamine tetraacetic acid), and clumps were removed by passing cells through a 30-μm prefilter. Cells were then centrifuged at 400 × *g* for 15 min. The cell pellet was suspended in MACS buffer (10^7^ cells in 80 μl), mixed with 20( μl of anti-CD14 MACS antibody-coated microbeads (Miltenyi Biotec), and incubated for 15 min at R°T. The cell suspension was applied to an LS-positive selection column that was previously washed with 1 ml of MACS buffer, and placed in a magnetic separation unit. The column was rinsed with 3.5 ml of MACS buffer, then removed from the magnetic separation unit, and positive bound cells were flushed with 2.5 ml of buffer.

### Set Up of Medium at Established pH Values

Culture medium at specific pH was obtained by adjusting the concentration of sodium bicarbonate according to the Henderson–Hasselbalch equation, as previously described (Avnet et al., 2013). To model acidic conditions, the pH of the medium was adjusted to 6.8. At different time-points and at the end-point of each experiment, the pH of the culture supernatants was measured to confirm the maintenance of the prefixed pH values during the incubation period, by using a micro-electrode (pH 301, HANNA Instruments). For the measurement of the pH of the culture supernatant, the medium was collected by using plastic tips and tubes that were pre-incubated at the same temperature and CO_2_ concentration of the incubator for at least an hour. Then, collected medium was centrifuged and, the pH value of the supernatant was measured at room temperature and CO_2_ conditions, as quick as possible, to avoid that the measurement could be affected by the changes in atmospheric temperature and CO_2_. pH value was obtained as an average of three sequential measurements for each of the three different samples.

### Collection of Cell Culture Supernatants

Conditioned medium of carcinoma cells: Conditioned medium (CM) of the carcinoma cell lines was used for the OC differentiation and for the Type I collagen degradation assays, as described in the next paragraphs. It was obtained from cultures seeded in T75 cell culture flasks and cultured at standard conditions. In particular, when 70% confluence was reached, cells were washed with PBS, and incubated with complete high-glucose DMEM. After 48 h, the supernatant was collected, centrifuged, and stored at −80°C until use.

Conditioned medium of hOB: CM of hOB was used for the functional assays with cells of the OC lineage (OC differentiation, Type I collagen degradation, and CD14^+^ cell migration assays), as described in the next paragraphs. It was obtained from acid-stressed or not-stressed hOB (CM hOB^*pH*^ ^6^^⋅^^8^ and CM hOB^*pH*7^^⋅^^4^, respectively) by seeding cells in T25 cell culture flasks (3 × 10^5^ cells/flask) in standard conditions. After adhesion, hOBs were washed with PBS and cultured for 20 h in α-MEM plus 0.1% FBS (low-serum medium). Cells were then washed with PBS, and incubated again with low-serum medium at pH 6.8 or 7.4 for 10 h. The medium was then replaced with low-serum medium at pH 7.4, and cells were incubated for an additional 48 h for all the conditions. At the end of the incubation, the supernatants were collected, centrifuged, and stored at −80°C until use.

To quantify the levels of IL-6, IL-8, or RANKL secreted from hOB by ELISA assay, as described in the next paragraph, we used two different hOB primary cell cultures: (a) for IL-6 and IL-8, we used the supernatant of the already differentiated cells: commercial hOB cells were seeded at standard conditions (3 × 10^4^ cells/well) in 24-well plates. After adhesion, in order to ensure low-serum adaptation, cells were washed with PBS and cultured in α-MEM plus 0.1% FBS (low-serum medium) for 20 h. Cells were then washed with PBS, and incubated with low-serum medium at pH 6.8 or at pH 7.4. After 24 h, the supernatants were collected, centrifuged, and stored at -80°C until use; (b) for RANKL, since commercial hOBs do not secrete detectable levels of RANKL, we used hOBs that were previously induced to differentiate from mesenchymal precursors: BM-MSCs were seeded (3 × 10^4^ cells/well) in 24-well plates at standard condition, and then cultured in osteogenic medium (complete α-MEM supplemented with 50 μg/ml of L-ascorbic acid 2-phosphate, 10^–8^ M dexamethasone, and 10 mM β-glycerophosphate, Sigma). After 10 days of culture, to ensure low-serum adaptation, cells were washed with PBS and cultured for 20 h in low-serum osteogenic medium. Cells were then washed with PBS, and incubated again with low-serum osteogenic medium at two different pH (6.8 or 7.4). After an additional 24 h, the supernatants were collected, centrifuged, and stored at −80°C until use.

### Osteoclast Differentiation Assays

Human OC precursors (PBMC) were isolated from buffy-coats of at least two healthy volunteers as previously described, and seeded in eight-well chamber slides. After 2 h of incubation at 37°C in a humidified 5% CO_2_ atmosphere, the medium was replaced, and the resulting mononuclear adherent precursors were cultured for 7 days with collected CM at a defined ratio depending on the specific experiment (see Table 1A), and compared with the respective negative control.

**TABLE 1A:** Description of the conditioned medium used in the different experiments.

**Figures**	**Conditions/treatments**	**Negative control**
Figure 1A	CM collected from the respective carcinoma cell culture **(A)**, mixed with OC complete medium **(B)**.	100% OC complete medium
	Ratio: **(A)** added with **(B)** at a ratio 1:4; 25%	
Figure 5A	CM collected from hOB cultures, pre-treated at different pH **(A)**, mixed with OC complete medium **(B)**.	Low-serum neutral alpha-MEM, mixed with **(B)** at a ratio 1:2; 50%.
	Ratio: **(A)** added with **(B)** at a ratio 1:2; 50%	
Figure 7B	CM collected from hOB cultures, pre-treated at different pH **(A)**, mixed with OC complete medium **(B)**. The final solution was also added or not with tocilizumab or with an anti-IL-8 antibody.	Low-serum neutral alpha-MEM, mixed with **(B)** at a ratio 1:2; 50%.
	Ratio: **(A)** added with **(B)** at a ratio 1:2; 50%	

For all the cell cultures, medium was changed every 3 or 4 days. When specifically mentioned, anti-IL-6 monoclonal antibody (TCZ, 100 μg/ml, Roche) or anti-IL-8 antibody (anti-IL-8 Ab, 5 μg/ml, R&D Systems) were added every 24 h. After 7 days of culture, cells were analyzed for TRACP expression (Acid Phosphatase, Leukocyte kit, Sigma-Aldrich), according to the manufacturer’s protocols, dark incubated with 2.25 μg/ml of Hoechst 33258 (Sigma-Aldrich) at RT for 10 min, and visualized with a Nikon Eclipse E800M fluorescence microscope (Nikon). TRACP-positive cells that showed more than three nuclei were considered as OC and counted. For assays, at least four replicates were performed.

### Osteoclast Activity: Type I Collagen Degradation

Testing the direct effect of acidic pH: hOC precursors (PBMC) isolated from buffy-coats of at least two healthy volunteers were seeded in 96-well plates coated with europium-conjugated type I collagen (Osteolyse Assay Kit, Human Collagen, Lonza). After 2 h from seeding, non-adherent cells were removed, and cells were cultured for 7 days in fresh complete high-glucose DMEM, added with 50 ng/ml of RANKL and 10 ng/ml of M-CSF (Peprotech) (differentiation medium) in order to obtain osteoclastogenesis. Then, medium was replaced with acidic or neutral differentiation medium, and cells were incubated for an additional 4 days. At the end-point, the cell supernatants were transferred into wells containing the fluorophore-releasing reagent. Fluorescence emission was immediately measured with a microplate reader (Infinite F200pro, Tecan) using an excitation wavelength of 340 nm and an emission wavelength of 615 nm. Data were expressed as relative fluorescence units (RFU), and hOC-mediated degradation of human bone collagen was considered to be directly proportional to the RFU recorded. In order to exclude the type I collagen degradation that was not the result of cell activity, we subtracted the signal obtained with cell-free acidic differentiation medium. All experiments were performed in quadruplicates.

Testing the effect of the cell secretome: Differentiated hOC were obtained after 7 days of incubation with differentiation medium, as described above. Then, the medium was replaced, and cells were incubated for an additional 4 days with the collected CM, at a defined ratio depending on the specific experiment (see Table 1B), and compared with the respective control.

**TABLE 1B:** Description of the conditioned medium used in the different experiments.

**Figures**	**Description of the used medium**	**Negative control**
Figure 1B	CM collected from the respective carcinoma cell culture **(A)**, mixed with OC complete medium **(B)**.	100% OC complete medium
	Ratio: **(A)** added with **(B)** at a ratio 1:4; 25%	
Figure 7C	CM collected from hOB cultures, pre-treated at different pH **(A)**, mixed with OC complete medium **(B)**. The final solution was also added or not added with tocilizumab or with an anti-IL-8 antibody.	Low-serum neutral alpha-MEM, mixed with (b) at a ratio 1:2; 50%.
	Ratio: **(A)** added with **(B)** at a ratio 1:2; 50%	

For the experiments with TCZ or anti-IL-8 Ab treatment, the CM of hOB were added or not with 100 μg/ml TCZ or with 5 μg/ml anti-IL-8 Ab every 24 h. At the end-point, the cell supernatants were transferred into wells containing the fluorophore releasing reagent. Fluorescence emission was immediately measured as above described. Data were expressed as relative fluorescence units (RFU) after blank subtraction, and hOC-mediated degradation of human bone collagen was considered directly proportional to the RFU recorded. For all assays, at least eight replicates were performed.

### Gene Expression

RNA isolation from carcinoma cells: Total RNA was extracted from cell cultures using TRIzol reagent (Invitrogen, Thermo Fisher Scientific) and reverse transcribed with MuLV reverse transcriptase (Applied Biosystems, Thermo Fisher Scientific). For carcinoma cell lines, RNA was isolated from semi-confluent cells. Cells were first plated in complete IMDM and incubated for 24 h in a standard incubator. Then, for an additional 24 h for normoxic condition (21% O_2_), cells were maintained in the same incubator, whereas for hypoxic conditions (1% O_2_), cells were transferred to a hypoxic Invivo2-400 incubator (Ruskin Technologies).

RNA isolation from human osteoblasts: For RNA isolation from hOB under acidic conditions, cells were first plated in complete D-MEM. After adhesion, to ensure low-serum adaptation, cells were washed with PBS, and pre-treated for 20 h with alpha-MEM plus 0.1% FBS (low-serum medium). Then, at time 0 (T0), after washing with PBS, cells were incubated with low-serum Alpha-MEM at pH 6.8. RNA was collected after 3 and 24 h of incubation.

Quantitative reverse transcription polymerization chain reaction (qRT-PCR) protocols and primers: qRT-PCR was performed by amplifying 1 μg of cDNA using the light cycler instrument and the universal probe library system (Roche Applied Science). Probes and primers were selected using a web-based assay design software (ProbeFinder, ^1^). Primer sequences and probes are listed in Table 2.

**TABLE 2:** Probe and primers.

**Gene**	**Full name**	**Accession number**	**Primers**	**Probe**
*ACTB*	Actin, beta	NM_001101.2	F = ccaccgcgagaagatga	64
			R = ccagaggcgtacagggatag	
HMBS	Hydroxymethylbilane synthase	NM_000190.3	F = tgtggtgggaaccagctc	26
			R = tgttgaggtttccccgaat	
*CA9*	Carbonic anhydrase 9	NM_001216.2	F = tgcctatgagcagttgctgt	73
			R = ccagtcctgggacctgagt	
*ATP6V1B2*	ATPase H + transporting V1 subunit B2	NM_001693.3	R = tggccgaagacttccttg	6
			F = ccgaaatgccagtctgaatc	
*ATP6V0C*	ATPase, H + transporting, lysosomal 16 kDa, V0 subunit c	NM_001694.2	R = ttcgtttttcgccgtcat	76
			F = ccactgggatgatggacttc	
ATP6V1G1	ATPase H = transporting V1 subunit G1	NM_004888.3	R = tcagtctcaggggattcagc	75
			F = tcagcctgagcttcttctttg	
M-CSF	Macrophage colony-stimulating factor	M64592.1	R = gcaagaactgcaacaacagc	19
			F = atcaggcttggtcaccacat	
TNF	Tumor necrosis factor	NM_000594.2	R = cagcctcttctccttcctgat	29
			F = gccagagggctgattagaga	
*IL-6*	Interleukin 6	NM_000600.3	F = gatgagtacaaaagtcctgatcca	40
			R = ctgcagccactggttctgt	
*IL-8/CXCL8*	C-X-C motif chemokine ligand 8	NM_000584.3	F = gagcactccataaggcacaaa	72
			R = atggttccttccggtggt	

Results were normalized to hydroxymethylbilane synthase (HMBS) or beta-actin (β-actin), according to the 2-ΔΔCT method (Avnet et al., 2017). For the experiments performed at pH 6.8, we used HMBS since we have previously demonstrated its stability under acidic conditions in human osteoblast-like cells (Lemma et al., 2018). Assay was repeated with four replicates.

### Quantification of the Extracellular Acidification Activity

Seahorse technology: Tumor cells were seeded on an XF microplate in complete IMDM, starved of glucose for 2 h, and then supplied with 2 g/L of D-glucose (Sigma-Aldrich). The complete ECAR analysis consisted of four stages: basal (without drugs), glycolysis induction (addition of 10 mM glucose), maximal glycolysis induction (addition of 2 μM oligomycin in order to impair oxidative phosphorylation), and glycolysis inhibition (addition of 100 mM 2-DG). The measurements of the proton production rates (PPRs) were obtained by using a Seahorse Extracellular Flux (XF-96) analyzer (Seahorse Bioscience). We considered PPR values obtained before the addition of D-glucose to be representative of non-glycolytic extracellular acidification (−glycEA), and those obtained after the addition of D-glucose to be representative of extracellular acidification derived from glycolysis (+glycEA). Data were normalized to the total protein content using the BCA protein assay (Pierce). This type of assay has a great variability since metabolic activity can change very quickly and is very sensitive to small variations in the microenvironmental conditions. Thus, for this test, the experiment was performed three times, and each condition had 15 intra-assay replicates (*n* = 45).

PHMed assay: As already previously described (Perut et al., 2014), 16 × 10^6^ cells were washed twice in pHMed solution [80% normal saline, 10% unbuffered RPMI-1640 (Sigma-Aldrich) and 10% FBS], and incubated in pHMed in suspension for 3 h at 37°C. Cells were then centrifuged (10 min at 500 × g), and supernatant was collected for extracellular pH (pHe) measurement. pHe was immediately quantified by a digital pH-meter (pH 301, HANNA Instruments). The experiment was repeated with six replicates for both breast and renal carcinoma cell lines.

### CD14^+^ Cell Migration Assay

CD14^+^ cells were isolated from PBMC after immunomagnetic separation, as previously described. The isolated cells were then suspended in 200 μl of alpha-MEM containing 0.1% BSA and seeded into Transwells with 8-μm pores (9 × 10^4^ cells/Transwell), in the upper compartment. In the lower compartment, 800 μl of alpha-MEM added with 5% of FBS at pH 7.4, or 800 μl of alpha-MEM added with 5% of FBS at pH 6.8, or 800 μl of CM hOB^*pH*^ ^7^^⋅^^4^, or 800 μl of CM hOB^*pH*^ ^6^^⋅^^8^ were placed. Cells were then incubated in 5% CO_2_ at 37°C, and allowed to migrate for 36 h. At the endpoint, migrated cells on the lower side were fixed in methanol, stained with crystal violet solution, and counted from nine random fields (20 × lens) in each well. The assay was repeated with six replicates.

### ELISA

Analysis of cell culture supernatants: hOB supernatants were analyzed for IL-6, IL-8, and RANKL content using Human IL-6 DuoSet ELISA kit, Human CXCL8/IL-8 Quantikine ELISA kit (RD System), and Human sRANK Ligand Standard ABTS ELISA Development kit (Peprotech), respectively. Normalization was performed with respect to the total protein content as measured by BCA protein assay. IL-6, IL-8, TRACP5b, and CTX were quantified in serum samples using the Human IL-6 DuoSet, Human CXCL8/IL-8 Quantikine (RD System), BoneTRAP^®^ (TRACP5b), and Serum Crosslaps^®^ (CTX-I) (Immunodiagnostic Systems) ELISA kits, respectively. The assay was repeated with four replicates.

Analysis of serum from patients: We collected blood samples from 29 patients at the time of BM diagnosis (20 from breast carcinomas, six from renal carcinomas, three from thyroid carcinomas) and that were enrolled since February 2014 to June 2017 by the Rizzoli Orthopedic Institute and the ANT foundation in Bologna, and by the Istituto Scientifico Romagnolo Per Lo Studio E La Cura Dei Tumori (IRST) in Meldola. The samples were collected at clinical presentation, before surgical treatment of the bone lesion, with signed informed consent and with institutional ethical committee approval (No. 0037602 of November 14, 2013). Blood samples were centrifuged (2,500 rpm, 8 min), and sera were aliquoted and stored at −80°C until the ELISA assays were performed.

### Animal Experiment

Animal experiment was conducted as a service by the Pharmatest Company (Turku, Finland), with the approval of the National Committee for Animal Experiments (license number ESAVI-2077-04 10 07-2014). Two 5- to 6-week-old female athymic nude mice (Hsd: Athymic nude, Foxn1nu; Envigo, Netherlands) were exposed to analgesic (Temgesic; buprenorphine, 0.1 mg/kg body weight) at least 30 min before the inoculation, and then injected with MDA-MB-231 (1 × 10^5^ cells in 20 μl of PBS) into the bone marrow of right proximal tibia. At 4 weeks after intratibial inoculation, the mice were sacrificed with CO_2_, and the death was confirmed by cervical dislocation. The tumor-bearing tibias were collected to 10% neutral-buffered formalin (NFB) for histological evaluation. Paraffin-embedded tumor-bearing tibias were prepared for histology. The tumor-bearing tibias were decalcified in ethylenediaminetetraacetic acid (EDTA) for 2 weeks. After de-calcification, the samples were dehydrated in an increasing series of ethanol concentrations, defatted in xylene, and embedded in paraffin (TEK III Paraffin Wax, Sakura, Netherlands). Then midsagittal 4-μm sections were obtained from each animal. For the histology, the sections were deparaffinized in xylene and rehydrated in a series of descending ethanol concentrations.

Sections were stained for hematoxylin and eosin (H&E-Orange G; Leica Biosystem and Sigma-Aldrich) for quantification of tumor area, and TRACP (Pararosaline; Sigma-Aldrich) for the identification of OC.

### Immunohistochemistry

Five-micrometer-thick tissue sections were mounted on a glass slide covered with 2% silane solution in acetone. After dewaxing in Citro Histoclear (Histo-Line Laboratories, Milano, Italy) and rehydration in ethanol, tissue sections were incubated first in a 3% hydrogen peroxide solution and then in a 2% bovine serum albumin solution in order to block the endogenous peroxidases and non-specific binding, respectively. Incubation with the rabbit anti-ATP6V1B2 or rabbit anti-LAMP2 (Sigma, Saint Louis, Missouri, United States) primary antibody followed. Tissue sections were then incubated with a biotinylated secondary antibody, covered with DAB, and counterstained with Mayer’s hematoxylin (EnVision FLEX, High pH Link visualization system, Agilent Technologies, Santa Clara, United States).

### Statistical Analysis

Because of the small number of observations, data were not considered to be normally distributed, and therefore, non-parametric tests were used. Statistical analysis was performed using GraphPad Prism 7 software (San Diego, CA, United States). For the difference between two groups, the Mann–Whitney *U*-test was used. For the correlations between serum levels of the different proteins analyzed, the Spearman’s rank test was used. Data were expressed as the mean ± standard error (SEM), and only *p*-values < 0.05 were considered for statistical significance.

## Results

### Carcinoma-Derived Extracellular Acidosis Enhances the Migration of OC Precursors

**FIGURE 1:**
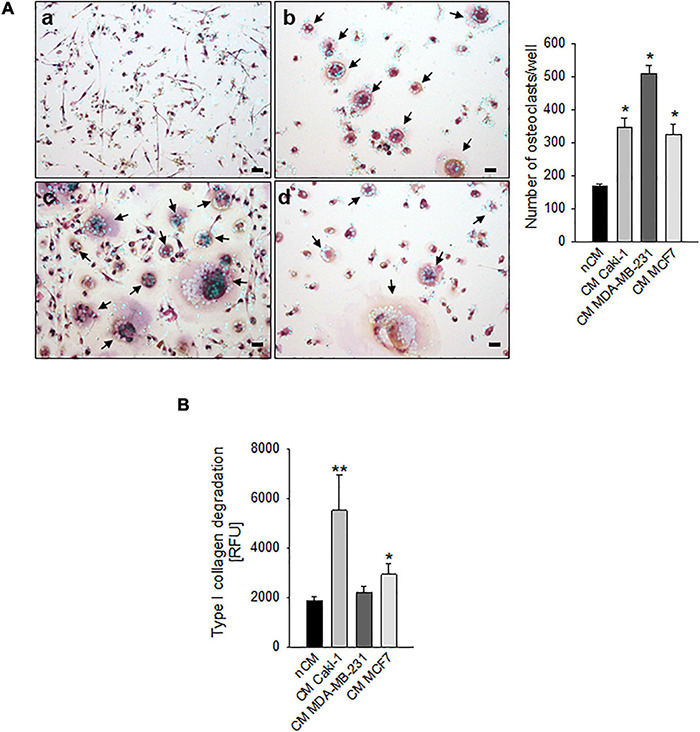
Carcinoma cells directly induced human osteoclast (hOC) differentiation and activity. **(A)** Graphs and representative images of the number of multinucleated TRACP^+^ hOC after the incubation with non-conditioned medium (nCM) (a) and with the conditioned medium (CM) of Caki-1 (b), MDA-MB-231 (c), and MCF7 (d) carcinoma cell lines. Mean ± SEM (*n* = 4, *p < 0.05 vs. nCM), scale bar 25 μm. Nuclei were counterstained with Hoechst 33258, black arrows indicate multinucleated TRACP^+^ hOC. **(B)** Type I collagen degradation activity of differentiated OC exposed to the conditioned medium (CM) of Caki-1, MDA-MB-231, and MCF7 carcinoma cell lines. Non-conditioned medium (nCM) was used as a negative control. Mean ± SEM (*n* = 8, *p < 0.05 and **p < 0.01 vs. nCM).

We considered different carcinomas that metastasize to bone. Most of them, such as the breast carcinoma cell lines MDA-MB-231 and MCF7, can directly promote differentiation of hOC (Figure 1A), whereas only MCF7 can induce a slight increase in OC activity (type I collagen degradation, Figure 1B) at *in vitro* standard culture condition and under physiological pH 7.4. Conversely, for renal carcinoma, we already reported that ACHN and CRBM-1990 cells induce hOC formation only indirectly, via the stimulation of stromal cells, such as endothelial cells (Cenni et al., 2006). This appeared not to be the case for Caki-1 cells that, in this study, directly induced OC differentiation and activity (Figures 1A,B).

**FIGURE 2:**
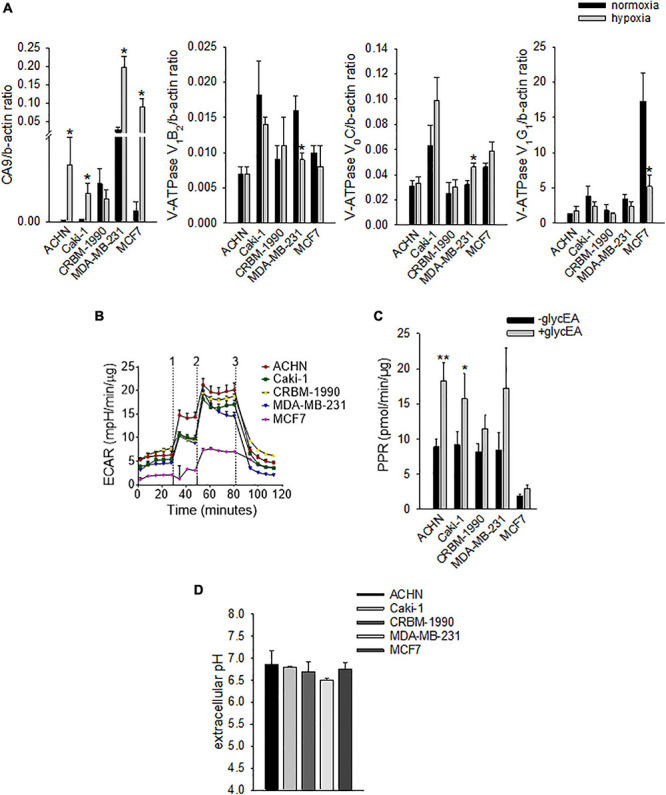
Carcinoma cells acidify the extracellular space by using different proton/ion transporters. **(A)** mRNA levels of CA9 and V-ATPase subunits (V_1_B_2_, V_0_C, and V_1_G_1_) under normoxia (21% O_2_) and hypoxia (1% O_2_). Mean ± SEM (*n* = 4 for CA9 and V_0_C, and *n* = 6 for V_1_B_2_ and V_1_G_1_, *p < 0.05). **(B)** Representative graph of extracellular acidification rate (ECAR); 1, 2, and 3 are the respective time-points for the injection of glucose, oligomycin, and 2-deoxy-D-glucose. **(C)** Extracellular acidification ability of carcinoma cell lines as determined by the measurement of the proton production rate (PPR); PPR values obtained before the addition of glucose represent non-glycolytic extracellular acidification (^–*glyc*^EA), in contrast to the values obtained after the addition of glucose (^+glyc^EA). Mean ± SEM (*n* = 45, *p < 0.05). **(D)** Extracellular acidification ability of carcinoma cell lines. The unbuffered pHMed solution was incubated with the respective cell suspension and measurement of extracellular pH was obtained by using a pH micro-electrode. Mean ± SEM (*n* = 4).

However, carcinoma cells may also modulate OC differentiation and activity through the acidification of the extracellular space that may derive both from hypoxia-dependent or -independent mechanisms. Thus, to confirm the ability of mammary and renal carcinoma cells to lower the extracellular pH, both under normoxia and hypoxia, we analyzed the mRNA expression of key regulators of intracellular and extracellular pH at different oxygen tension (Figure 2A).

**FIGURE 3:**
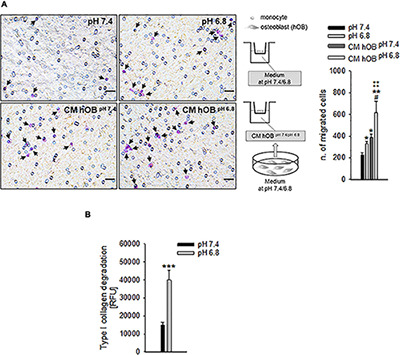
The recruitment of OC precursors and the activity of OC are enhanced by extracellular acidosis via different mechanisms. **(A)** CD14^+^ cells were seeded on the upper compartment of a Transwell system, and acidic or neutral medium (pH 6.8 and pH 7.4, respectively), or the conditioned medium of hOB treated or not treated with acidic medium (CM hOB^*pH*^ ^6^^⋅^^8^ and CM hOB^*pH*7^^⋅^^4^, respectively) were added to the lower compartment; after 36 h of exposure to the specific chemotactic stimulus, migrated cells were counted. On the left, representative images of CD14^+^ migrated cells stained with crystal violet (black arrows), scale bar 25 μm; on the right, scheme of the experiment and graph of the counted migrated cells, mean ± SEM (*n* = 6, *p < 0.05 and **p < 0.01 vs. pH 7.4, ^#^*p* < 0.05 vs. CM hOB^*pH*^ ^7^^⋅^^4^, ^‡‡^*p* < 0.01 vs. pH 6.8). **(B)** Type I collagen degradation activity of differentiated OC at acidic versus neutral condition, mean ± SEM (*n* = 8, ***p < 0.001).

Specifically, we analyzed the mRNA levels of the proton extruders carbonic anhydrase 9 (CA9) and V-ATPase. We found that renal carcinoma cell lines expressed CA9, V_1_B_2_, V_0_C, and V_1_G_1_ subunits of V-ATPase that were confirmed also in breast carcinoma cell lines. Furthermore, CA9 expression was significantly increased under reduced oxygen conditions with respect to normoxia (*p* = 0.0209 for MDA-MB-231 and ACHN, *p* = 0.0495 for MCF7, *p* = 0.0339 for Caki-1, Figure 2A). In contrast, among the different V-ATPase subunits, only for the V_0_C, we observed a trend of hypoxia-induced increased expression that was significant for the MDA-MB-231 cell line (*p* = 0.0433, Figure 2A). By using the Seahorse analyzer, we then directly quantified the acidification ability of both mammary and renal carcinoma cells (namely, the measurement of proton production rate, PPR) resulting from the glycolytic metabolism (+glycEA) or, independently, to glycolysis (−glycEA) (Figures 2B,C). Notably, all the examined carcinoma cell lines strongly acidified the extracellular space, and we still detected a meaningful secretion of protons in the -glycEA condition, except for MCF7 breast carcinoma cells (Figure 2C). We further confirmed this finding also by measuring the supernatant of the unbuffered cell cultures through the use of a micro-electrode. Although less precise, this approach enlightened that, *in vitro*, carcinoma cells acidified the extracellular fluid to an average pH value of 6.72 ± 0.06 (Figure 2D). Then, we investigated the effects of acidosis on cells of the BM microenvironment by using a medium buffered at pH 6.8 to mimic the acid supernatant of carcinoma cells in all the following experimental protocols. We selected the pH value that corresponded to the average pH of the supernatant of the carcinoma cell lines included in this study (Figure 2D, pHMed assay) that was also the same pH value of the tumor microenvironment, as assessed in a model of breast carcinoma, assuming that the intratumoral pH of BM deriving from breast cancer may well correspond to that of primary lesions (Longo et al., 2016). To dissect the role of tumor-derived extracellular acidification, we used fresh acid medium instead of the acidified supernatant of carcinoma cells, thereby avoiding other confounding factors like the secretion of paracrine molecules, including tumor-derived growth factors. Interestingly, we found that low pH directly enhances the recruitment of human CD14^+^ monocytes (Figure 3A, pH 6.8 vs. 7.4, *p* = 0.025). The incubation with acidic medium also significantly enhanced type I collagen degradation in cultured OC (Figure 3B).

**FIGURE 4:**
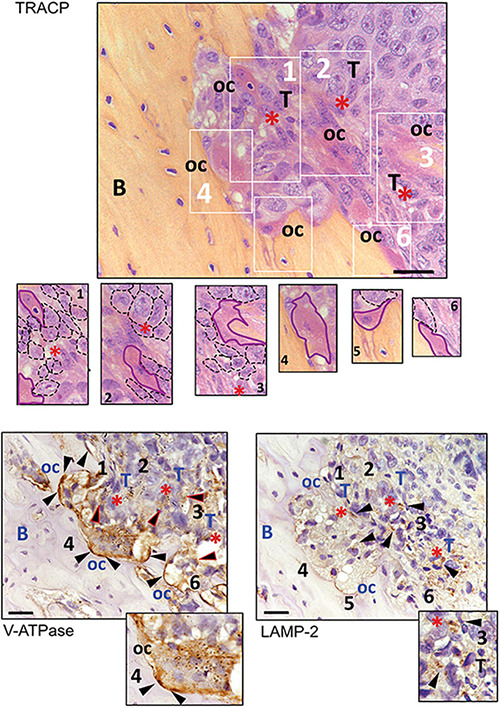
Immunostaining for TRACP, V_1_B_2_ V-ATPase, and LAMP2 in osteolytic area of bone metastasis (BM) xenografts. Representative pictures. In the upper panel, TRACP staining (bordeaux) enlighten the presence of multinucleated osteoclasts (nuclei were counterstained with Mayer’s hematoxylin). At the bottom, V-ATPase and LAMP2 staining of subsequent sections of the same sample (B, bone; oc, osteoclasts; T, area rich in tumor cells; red asterisks, supposed acidic area since they showed LAMP2-positive carcinoma cells; black triangles, positive signal for the respective antigen). In the image corresponding to TRACP staining, six different areas, identified with white numbers and rectangles, are also shown at higher magnification (osteoclasts have been contoured with continuous purple lines, whereas cancer cells were contoured with dark dotted lines). The same numbers have been used to identify the same area of the tumor in the subsequent tissue sections. Both osteoclasts and tumor cells showed to be positive for V-ATPase, although to a lower degree in tumor cells (triangles with red perimeters). Furthermore, the area with LAMP2 staining in carcinoma cells appeared to be very close to actively resorbing bone (scale bar 10 μM).

To confirm the close relationship between OC and acidifying carcinoma cells, we developed a xenograft model of BM by intra-tibial injection of MDA-MB-231 cells (Salerno et al., 2010; Di Pompo et al., 2017). We used MDA-MB-231 cells since they are derived from a breast carcinoma that, in human patients, causes osteolytic lesions more frequently than other carcinomas (Weilbaecher et al., 2011) and because, adversely to MCF7 cells, they can develop osteolytic bone metastases *in vivo* (Walenta et al., 2001; Robey et al., 2008). Moreover, we previously demonstrated that MDA-MB-231 cells have a Warburg phenotype characterized by an increased NADH/NAD+ ratio, GLUT1 expression, glycolytic rate, and, as a result, increased acidification in the extracellular microenvironment. On the contrary, the metabolism of the less-aggressive breast carcinoma cells MCF7 is mainly based on oxidative phosphorylation (Lemma et al., 2017). We then considered V-ATPase and LAMP2 expression as indirect markers of acidifying tumor cells and acidified tumor area (Avnet et al., 2013, 2019a; Damaghi et al., 2015). In the tumor sections obtained from this model, we observed the presence of actively resorbing OC that were identified by the staining for TRACP activity and V_1_B_2_ V-ATPase strong signals, with particular regard to the resorbing cell side that attached to the bone (Figure 4). As expected, the V-ATPase subunit was also expressed by carcinoma cells. In the tumor area in which carcinoma cells are coupled with OC, we also observed LAMP2 staining in cancer cells (Figure 4), indicating that those areas that are characterized by the presence of actively resorbing OC were associated with acidifying carcinoma cells.

### Extracellular Acidosis Feeds Osteolysis by Specific OB-OC Paracrine and Inflammatory Circuits

**FIGURE 5:**
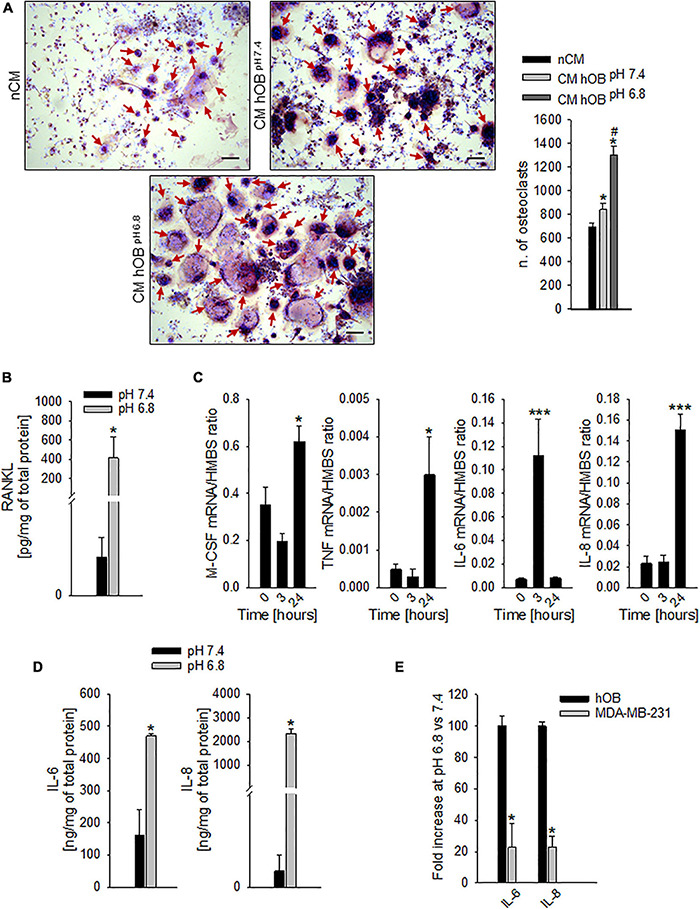
Extracellular acidosis promotes the secretion of pro-osteoclastogenic mediators from osteoblasts (OB). **(A)** On the right, graph of N. of TRACP+ and multinucleated OC obtained from PBMC, after 7 days of incubation with the CM hOB^*pH*^ ^6^^⋅^^8^, or the CM hOB^*pH*^ ^7^^⋅^^4^. The non-conditioned medium (nCM) was used as negative control. Mean ± SEM (*n* = 4, *p < 0.05 vs. nCM; #*p* < 0.05 vs. CM hOB^*pH*^ ^7^^⋅^^4^). On the left, representative images; scale bar 100 μm (red arrows show multinucleated TRACP + OC). **(B)** Levels of secreted free RANKL protein as detected by specific ELISA in hOB supernatant after incubation with acid or neutral medium. Mean ± SEM (*n* = 4, *p < 0.05). **(C)** Increase over the time of mRNA expression levels of M-CSF, TNF-α (TNF), IL-6, and IL-8 in hOB cultures, after the exposure to extracellular acidosis. Mean ± SEM (*n* = 4, *p < 0.05 and ***p < 0.001 vs. Time 0). **(D)** Protein levels of IL-6 and IL-8 by ELISA in hOB supernatant after incubation with acid or neutral medium (pH 6.8 and 7.4, respectively). Mean ± SEM (*n* = 4, *p < 0.05). **(E)** mRNA expression of IL-6 and IL-8 in MDA-MB-231 cells with respect to hOB exposed for 3 h to pH 6.8 (= K, 100%).

In addition to a direct effect, we speculated that extracellular acidosis indirectly recruits hOC precursors by inducing a chemotactic secretome in human OB (hOB). As a confirmation, when we used the conditioned medium of acid-stressed hOB (CM hOB^*pH*6^^⋅^^8^), the migratory activity of CD14^+^ monocytes was significantly higher compared with conditioned media from hOB^*pH*7^^⋅^^4^ or to acidic medium itself (Figure 3A, *p* = 0.0062). Notably, acid-stimulated hOB also indirectly promoted OC differentiation as CM hOB^*pH*6^^⋅^^8^ significantly increased the number of mature hOC in respect with CM of not acid-stressed human hOB (CM hOB^*pH*7^^⋅^^4^) (*p* = 0.0209, Figure 5A).

To go deeper inside the identification of pro-osteoclastogenic growth factors that are released by acid-stimulated hOB, we found that exposure to acidic medium significantly increased the level of secreted free RANKL protein, with respect to neutral conditions (Figure 5B, *p* = 0.0339), and, after 24 h, the level of the mRNA expression of the macrophage colony-stimulating factor (M-CSF) and TNF-α (*p* = 0.0274 and *p* = 0.0205 vs. time 0, respectively, Figure 5C). Similarly, also the mRNA transcription of the pro-inflammatory cytokines IL-6 and IL-8 was increased (at 3 and 24 h for IL-6 and IL-8, respectively, *p* = 0.0008 for both IL-6 and IL-8, Figure 5C). Protein analysis confirmed the augmented secretion of the two pro-inflammatory cytokines at low pH conditions (*p* = 0.0209 for both IL-6 and IL-8, Figure 5D). Notably, the acid-stimulated levels of expression of the inflammatory mediators and pro-osteoclastogenic factors IL-6 and IL-8 were significantly higher in hOB than in the tumor cell line MDA-MB-231 (*p* = 0.0339, Figure 5E).

**FIGURE 6:**
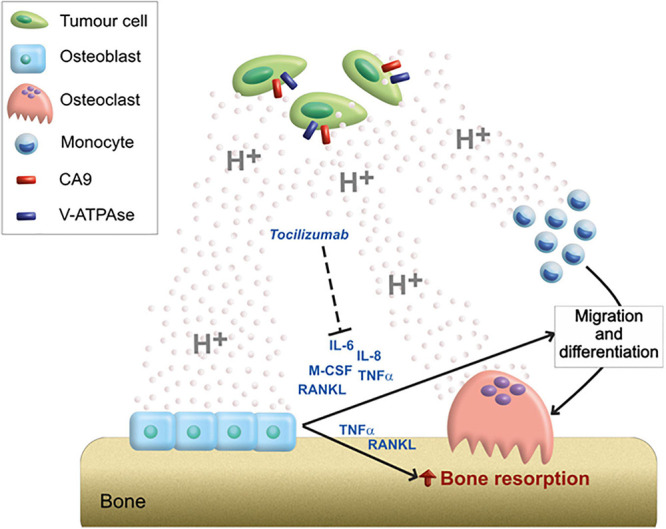
Graphical scheme of the mechanistic model proposed for the role of acidosis on the bone remodeling unbalance. Tumor cells acidifying the extracellular environment through the activity of CA9 and V-ATPase; acidosis can directly stimulate the migration of OC precursors and activate bone-resorbing OC; however, the high amount of protons in the extracellular space can also induce the release of a pro-osteoclastigenic secretome by OB (IL-6, IL-8, M-CSF, TNFα, and RANKL). The paracrine mediators secreted by acidosis-induced OB, might, in turn, further exacerbate the OB–OC coupling responsible for osteolysis. Among them, the targeting of IL-6 by the anti-IL-6R antibody (Tocilizumab) blocks the acidosis-promoted and osteoblast-mediated induction of OC differentiation and activity.

Our preliminary *in vitro* data suggested that an acidic microenvironment, in addition to directly promote the migration of OC precursors and the bone resorption activity of mature OC, activates an inflammatory and pro-osteoclastogenic secretome in OB. In more detail, we speculated that the acidic microenvironment of BM further exacerbates osteolysis, both directly and by regulating the paracrine interaction between OB and OC, in favor of OC differentiation and OC-mediated bone resorption (Figure 6).

### Circulating Levels of Inflammatory Cytokines IL-8 and IL-6 and Their Correlation With Osteolysis

By analyzing circulating markers in human patients, we then indirectly verified whether the inflammatory cytokines that are possibly released by locally acid-stimulated osteoblasts in the BM microenvironment are also released at a systemic level. If this hypothesis is correct, the circulating levels of IL-6 and IL-8 may reflect a local increase in the respective cytokines in the bone tissue and may parallel the levels of osteolysis in human patients. As a first step, we validated the tartrate-resistant acid phosphatase 5b (TRACP5b) as a specific and sensitive marker of OC activity (Halleen et al., 2000, 2001; Avnet et al., 2008; Savarino et al., 2010) in a continuous series (*n* = 29) of patients with osteolytic BM from breast, renal, and thyroid carcinoma (Table 3).

**TABLE 3:** Clinical features of patients with BM.

**Patient**	**Age**	**Sex**	**Type of carcinoma**	**Number of BM**	**TRACP5b serum level (U/L)**	**CPR (mg/dl)**
1	59	M	Renal	Multiple	0.35	1.81
2	84	F	Renal	Multiple	0.48	6.85
3	69	F	Renal	Solitary (humerus)	0.67	0.24
4	59	F	Mammary	Multiple	0.23	0.41
5	59	F	Mammary	Solitary (femur)	0.64	0.46
6	65	F	Mammary	Multiple	0.75	n.d.
7	45	F	Mammary	Solitary (scapula)	0.42	0.1
8	79	F	Mammary	n.d.	1.85	n.d.
9	73	F	aMmmary	Multiple	10.11	n.d.
10	56	M	Thyroid	Multiple	1.06	0.13
11	81	F	Mammary	Solitary (femur)	4.23	1.12
12	67	F	Mammary	Multiple	4.23	0.35
13	52	F	Mammary	Multiple	4.45	0.39
14	42	F	Mammary	Solitary (femur)	1.32	0.14
15	48	M	Thyroid	Solitary (pelvis)	3.29	0.18
16	79	F	Mammary	Solitary (humerus)	3.43	0.24
17	56	F	Mammary	Solitary (humerus)	2.573	1.26
18	77	F	Thyroid	Multiple	3.38	1.46
19	60	M	Renal	Multiple	0.95	n.d.
20	66	F	Mammary	Multiple	4.56	n.d.
21	38	F	Mammary	Multiple	8.506	n.d.
22	50	F	Mammary	Solitary (spine)	11.08	n.d.
23	59	M	Renal	Multiple	5.64	n.d.
24	47	F	Mammary	Multiple	1.82	n.d.
25	58	F	Mammary	Multiple	2.69	n.d.
26	71	F	Mammary	Multiple	14.04	n.d.
27	62	F	Mammary	Solitary (skull)	3.29	n.d.
28	72	F	Renal	Multiple	5.15	n.d.
29	64	F	Mammary	Multiple	3.23	5,75

*n.d., not determined.*

**FIGURE 7:**
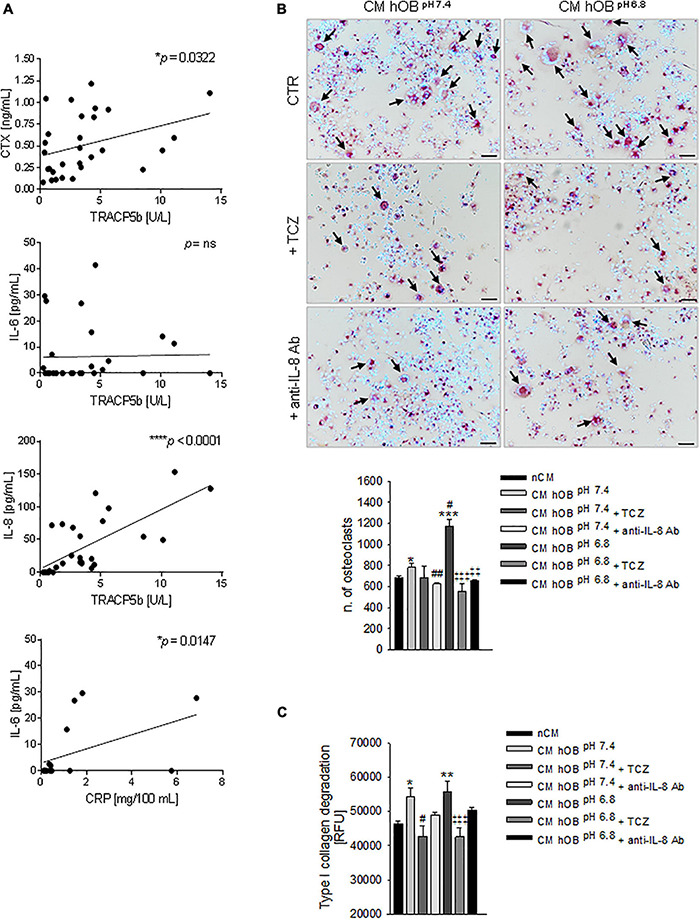
Interleukin 6 (IL-6) and Interleukin 8 (IL-8) and osteolysis in BM. **(A)** Correlation in BM patients between serum levels of CTX and TRACP5b, IL-8, and TRACP5b, IL-6 and TRACP5b, and IL-6 and C-reactive protein (CRP). Mean ± SEM (*n* = 29, *p < 0.05, ***p < 0.0001; *n* = 16, *p < 0.05 for IL-6 and CRP correlation). **(B)** On the left panel, representative images of TRACP positive multinucleated OC after 7 days of treatment with CM hOB^*pH*^ ^6^^⋅^^8^ or CM hOB^*pH*^ ^7^^⋅^^4^ added or not with the anti-IL-6 antibody, tocilizumab (TCZ) and the anti-IL-8 antibody (anti-IL-8 Ab) (scale bar 50 μm). On the right panel, graph of the quantification of n. of mature OC. Mean ± SEM (*n* = 8, *p < 0.05 and ***p < 0.001 versus nCM, ^#^*p* < 0.05 and ^##^*p* < 0.05 versus CM hOB^*pH*^ ^7^^⋅^^4^; ^‡‡^*p* < 0.01 and ^‡‡‡^*p* < 0.001 versus CM hOB^*pH*^ ^6^.^8^). **(C)** Bone resorption activity of OC treated with the same conditions of **(B)**, and measured as type I collagen degradation. In this case, TCZ and anti-IL-8 Ab were added starting from day 7, after that mature OCs were formed. Mean ± SEM (*n* = 15, *p < 0.05 and **p < 0.01 versus nCM, #*p* < 0.05 versus CM hOB^*pH*^ ^7^^⋅^^4^; ^‡‡‡^*p* < 0.001 versus CM hOB^*pH*^ ^6^.^8^).

In this series of patients, we confirmed a significant correlation (*p* = 0.0322) between the serum levels of the two bone resorption-related markers, the TRACP5b and the cross-linked telopeptide of type I collagen (CTX) (Figure 7A).

Subsequently, by ELISA assays, we found a highly significant correlation between TRACP5b and IL-8 (*p* < 0.0001) and a lack of correlation with IL-6 (Figure 7A). On the contrary, IL-6 correlated with C-reactive protein (CRP) (Figure 7A).

Notably, in our *in vitro* model, IL-8 was more highly expressed in acid-stressed hOB with respect to IL-6 (2,332.53 ± 176.41 versus 469.78 ± 7.08 ng/mg of total protein, respectively, Figure 5D).

### Targeting IL-6 and IL-8

Based on the evidence obtained, IL-6 and IL-8 are possible regulators of the proposed mechanistic model (see Figure 6). We then sought to investigate the effectiveness of using the neutralizing anti-IL-6R antibody TCZ and anti-IL-8 antibody (Figure 6) that is already clinically used to treat different diseases (Kang et al., 2015). When OC precursors were cultured in the presence of CM hOB^*pH*6^^⋅^^8^ that was added with TCZ or with an anti-IL-8 antibody, hOC differentiation significantly decreased (*p* = 0.0008 and *p* = 0.0066, respectively; Figure 7B). Conversely, no significant inhibitory effects were observed after exposure to CM hOB^*pH*7^^⋅^^4^, added with TCZ. These data suggest that the pro-osteoclastogenic effect exerted by the acid-induced secretome of OB specifically relays on IL-6 and IL-8 secretion, while the stimulatory effect of CM hOB^*pH*7^^⋅^^4^ is possibly mediated by factors other than IL-6. Based on our data, IL-8 may be one of these mediators since neutralizing IL-8 strongly affected osteoclastogenesis after exposure to both neutral and acid-induced secretome of OB (*p* = 0.0065 and *p* = 0.0066, respectively). Regarding OC resorption activity, only TCZ significantly inhibited type I collagen degradation induced by CM hOB at both pH (*p* = 0.0184 for CM hOB^*pH*7^^⋅^^4^ and *p* = 0.0007 for CM hOB^*pH*6^^⋅^^8^). However, the percentage of inhibition obtained with TCZ treatment was comparable between CM hOB^*pH*6^^⋅^^8^ and CM hOB^*pH*7^^⋅^^4^ (23.62 versus 21.30%, respectively, Figure 7C), suggesting that the acid-induced secretome of hOB is more effective in promoting OC differentiation rather than OC activity.

## Discussion

We previously demonstrated that BM is associated with an acidic microenvironment that, through the release of inflammatory mediators and neurotrophic factors, is responsible for the induction of pain (Di Pompo et al., 2017). However, extracellular acidosis in the BM microenvironment also interferes with bone remodeling, thereby contributing to altering the quality and structure of bone and allowing tumor local invasion. For the identification of novel therapeutic targets, it is crucial to deeply investigate all the key steps that are behind this mechanism.

In this study, as already shown by previous authors (Bendre et al., 2002; Liverani et al., 2014; Liang et al., 2019; Spadazzi et al., 2019), we first confirmed the ability of breast carcinoma cells and of the Caki-1 renal carcinoma cells to induce OC differentiation and activity, although this effect was obtained at a different extent according to the specific cell line used. These results are in agreement with the previous literature showing that carcinoma cells can secrete several factors that can directly induce OC differentiation via RANKL-independent mechanisms. Of these growth factors, TNF-α, IL-8, TGF-β, M-CSF, and VEGF can have a direct effect on OC differentiation but not on OC activity (Wang et al., 2020). Of note, MDA-MB-231 cells that in our study showed the greatest effect are known to secrete very high levels of M-CSF, also higher than MCF7 cells (Lu et al., 2019). We also found that among the carcinoma cell lines examined, Caki-1 cells can directly and strongly promote OC activity, whereas MCF7 showed a very low, although significant effect, and MDA-MB-231 cells were not able to directly induce OC-mediated collagen type I degradation. Also in this case, in the absence of extracellular acidification (this experiment was performed in buffered conditions), this phenotype may be explained only by the direct secretion of specific growth factors or miRNA that can directly stimulate OC resorption. The most important growth factors that can promote this effect are RANKL, and at a lower extent, IL-6 and IL-8. However, RANKL is not expressed by Caki-1 cells (Mikami et al., 2009), and IL-6 and IL-8 can be expressed mainly in induced-Caki1 cells (Pajdzik et al., 2020), and thus, the mechanism has yet to be identified.

We then confirmed that both breast and renal carcinoma cells express CA9 and different subunits of the proton pump V-ATPase (You et al., 2009; Di Pompo et al., 2017) and that CA9 expression was augmented when cells were exposed to hypoxic conditions. Hypoxia has been already associated with BM progression (Cox et al., 2018) and with increased extracellular acidosis (Pillai et al., 2019). In contrast, among the different V-ATPase subunits, only the V0C (ATP6V0C) appeared to be affected by low oxygen tension, especially for the MDA-MB-231 bone-metastatic cell line. The CAs are a family of enzymes that reversibly catalyze CO_2_ hydration to H^+^ and HCO_3_^–^ (Supuran, 2008). In particular, CA9 expression has been strongly associated with cancer aggressiveness and H^+^ fluxes versus the extracellular space, especially under hypoxia (Swietach et al., 2007). Regarding the pore-forming subunit V0C of vacuolar-H^+^-ATPase, it is interesting to note that it is strongly expressed in pancreatic carcinoma (Ohta et al., 1996) and highly invasive esophageal cancer cells (Son et al., 2019). According to data of Son et al., in these cells, ATP6V0C regulates glycolysis via pyruvate kinase isoform M2 (PKM2) through its recruitment at hypoxia response element (HRE) sites, in the lactate dehydrogenase A (LDHA) gene. Thus, although it needs to be confirmed with further experiments, according to these data, it is possible to speculate that in our carcinoma models, oxygen tension similarly modulates ATP6V0C activity.

We then verified the acidification ability of breast and renal carcinoma cells by directly measuring their proton production rate, and we found a significant extracellular secretion of protons as a consequence of both glycolytic-dependent and -independent mechanisms for all the tested carcinoma cell lines, but the less aggressive estrogen receptor-positive MCF7 breast carcinoma cells (Kirschmann et al., 1999; Wright et al., 2016). These results suggest that the acidification activity of carcinoma cells occurs also under basal oxidative metabolism and can be further enhanced, either by Warburg effect or by hypoxia-induced glycolysis.

In the context of BM, the resulting excess of protons in the interstitial spaces of the tumor may directly result in significant impairment of bone formation to the benefit of tumor-induced bone destruction. In more detail, carcinoma cells promote osteolysis, not only via the secretion of pro-osteoclastogenic factors but also by the acidification of the BM microenvironment. To confirm that tumor-derived acidosis has an activity on osteolysis that is independent of tumor-derived growth factors in *in vitro* experiments, we mimicked the tumor-derived acidosis by using a medium at a preset acid pH that was adjusted using a bicarbonate-CO_2_ buffering system. In more detail, we wanted to avoid confounding factors like the secretion of tumor-derived growth factors (i.e., RANKL or PTHrP) that would overlap or synergize with the effects derived from the exposure to extracellular acidosis. As a preset pH value, we chose pH 6.8 since it was the average pH value measured in the carcinoma cell supernatants as assessed by the *in vitro* pHMed assay and because it corresponds to the intratumoral pH value that was detected by MRI-CEST analysis in a subcutaneous model of breast carcinoma (Longo et al., 2016). Therefore, we assumed that the extracellular pH resulting from the altered metabolism of primary carcinoma cells correspond to that within BM.

The first step of the osteolytic process is the enrollment of OC precursors from the bloodstream at the site of the bone metastatic niche, and to broaden our knowledge on this topic, for the first time, we found that pH around 6.5 is sufficient *per se* to attract OC precursors. At the same time, it is well established that a low pH augments OC bone resorption activity (Krieger et al., 2004; Arnett, 2010), does not allow the differentiation of stem cells toward the OB lineage (Massa et al., 2017), and strongly affects bone mineralization (Brandao-Burch et al., 2005), thereby inducing an unbalance of the bone remodeling process in favor of osteolysis. As a demonstration, here, we showed *in vivo*, in a xenograft model of osteolytic BM from breast carcinoma, that acidifying carcinoma cells expressing LAMP-2 and V-ATPase are closely associated with actively resorbing OC.

The direct stimulation of OC differentiation and activity or the direct inhibition of osteoblast differentiation and activity may not be the sole mechanisms of tumor-derived acidosis to promote an osteolytic phenotype. According to our results, chemoattraction of OC precursors appeared to be even higher when we used the secretome of acid-stressed OB. The same secretome also heavily committed the undifferentiated monocytes versus the OC lineage. This effect was mediated by the release of well-known pro-osteoclastogenic factors, including RANKL and M-CSF, in addition to inflammatory modulators, like TNF, IL-6, and above the others, IL-8. In agreement with our report, a few previous studies have shown high levels of IL-6 or IL-8 in the serum of BM patients (Benoy et al., 2004; Di Pompo et al., 2017; Noman et al., 2017) and the attitude of stressed OB to secrete the same inflammatory cytokines (Marriott et al., 2004; Dapunt et al., 2016). Strikingly, IL-6 and IL-8 expression in acid-conditioned OB was higher than those of carcinoma cells. In a previous study, we demonstrated the same increase in IL-6 and IL-8 in OB precursors, the MSC, after the exposure to acidosis. Nevertheless, by comparing the relative concentrations, it appeared that the secretion of IL-8 was five times more in OB than in MSC (2,436.118 vs 534.653 ng/mg of total protein) after exposure to pH 6.8, whereas IL-6 levels appeared to be of the same order of magnitude between the two cell types (Avnet et al., 2017; Di Pompo et al., 2017). In the same study, we also demonstrated that IL-6 and IL-8 increased secretion was due to the activation of the NF-kB inflammatory pathway. It is, therefore, assumable that this mechanism is similarly primed in well-differentiated cells of the same lineage, like OB. Indeed, acidosis can activate stress signaling cascades, such as the NF-kB and the activator protein-1 pathways, in different cell models (Xie and Huang, 2003; Avnet et al., 2019a). In turn, secreted IL-6 and IL-8 by acid-stimulated OB may favor tumor progression in many different ways. A number of studies have shown IL-6 and its major effector STAT3 as pro-tumorigenic mediators in many cancers, including melanoma, breast, lung, colon, prostate, ovarian, and hematological cancers, and to be associated with OB–OC coupling via notch signaling (Sethi et al., 2011). IL-6 serum levels have also been shown to be significantly elevated in lung and breast cancer patients and associated with poor prognosis (Hodge et al., 2005). Both IL-6 and IL-8 are well-recognized pro-osteoclastogenic cytokines (Amarasekara et al., 2018). In particular, IL-8 induces RANKL-independent OC formation and activity (Bendre et al., 2003). Indeed, at the bone metastatic site, IL-6 indirectly contributes to OC differentiation and activation by increasing RANKL expression in OB via the JAK/STAT-3 signaling pathway (Palmqvist et al., 2002), thereby further promoting tumor local aggressiveness. Similarly, also IL-8 overexpression correlates with increasing tumor grade and metastasis in breast and prostate carcinoma and favors tumor cell motility, proliferation, and angiogenesis (Liu et al., 2016). Furthermore, IL-8 expression is induced by prolonged hypoxia and decreased intracellular pH in pancreatic and prostate cancer cells, and a few reports mentioned this cytokine as a pro-osteoclastogenic factor (Bendre et al., 2003).

In this study, we showed the very strong correlation between osteolysis, as detected by circulating concentration of TRACP5b and IL-8 in BM patients. TRACP5b is a specific index of bone resorption, and an accurate marker of OC number since it is released from OC into the blood circulation, together with matrix degradation products, during the bone resorption process through the basolateral membrane (Avnet et al., 2008; Savarino et al., 2010; Urakawa et al., 2020). However, in these patients, we could not directly prove that the release of these inflammatory mediators is derived from the cancer-induced bone lesion or from intratumoral acidosis. Nevertheless, it is noteworthy that the same cytokine that was secreted *in vitro* by acid-stressed hOB at the highest level, among the others, is also the same cytokine that is higher in patients with the highest level of TRACP5b and that these two biomarkers correlated at a very high significant level. Vice versa, the correlation between IL-6 and TRAC5b was weaker, possibly because IL-6 serum levels are strongly affected by the presence of systemic inflammation that is associated with high IL-6 circulating levels (Tanaka et al., 2014). On this regard, it is noteworthy that we recently found that C-reactive protein, a well-known marker of inflammation, highly significantly correlates with a poor prognosis rather than with BM (Errani et al., 2021), as also demonstrated in sarcomas (Nakamura et al., 2017). In conclusion, we cannot directly demonstrate that the circulating IL-8 is derived from acid-stressed cells of the OB lineage in BM patients. However, based on our data, we can speculate that, in these patients, osteolysis is correlated with the secretion of IL-8 that, in turn, might be derived from local acidic-stress cells of the OB lineage at the bone metastatic site.

Overall, our results suggest a crucial role for inflammatory cytokines that are stimulated by tumor acidosis in the bone microenvironment, and that in the context of BM alter the delicate balance of bone remodeling to the benefit of tumor progression. In this case, bone inflammation that is originated and maintained by tumor-associated acidosis concurs to exacerbate OB-mediated osteolysis. This is not a completely novel concept as it has already been demonstrated in a non-malignant disease that the inflammatory response can reduce local pH to 5.5 and that the acidic pH is itself damaging to the surrounding tissues also by stimulating new inflammatory reactions and prolonging the healing process (Morone et al., 2020). Indeed, at this point, it is to be acknowledged that acidosis does not only occur in tumors but also in several additional disease conditions, including inflammation, bone fractures (Dong et al., 2013), and intervertebral disc degeneration (Urban, 2002). Thus, the acid-induced mechanism of uncoupling that we observed between osteoblasts and OC may occur also in these conditions.

There are several concluded or ongoing clinical trials for the use of drugs for IL-8 and IL-6 targeting, or IL-6 receptor (IL6R) targeting, in patients with different diseases, including solid tumors and metastatic cancers (NCT04347226, NCT02536469, are some examples from^2^). HuMax-IL-8 or BMS-986253 for IL-8, and TCZ or siltuximab, or CNTO328, or olokizumab/CDP6038 for IL-6 and IL-6R are the most used drugs (Bilusic et al., 2019). The number of these clinical studies is exponentially increasing due to the current COVID-19 (SARS-CoV-2) pandemic situation. Indeed, these drugs are considered as potentially effective also against the COVID-19-induced cytokine storm in oncologic patients (Ragab et al., 2020). To date, the total number of concluded and active clinical studies on the use of anti-IL-8 drugs in cancer patients is two, whereas for anti-IL-6 drugs is 20. However, none of them is focused on BM, and the success of these treatments in reducing the impact of BM and the underlying mechanisms have to be proven yet. We next focused on the investigation of TCZ and anti-IL-8 antibody effects in our model. As a starting point for our experimental approach, we referred to a study that used TCZ on a xenograft model of BM injected with MDA-MB-231 cells (Wakabayashi et al., 2018). The treatment reduced BM as revealed by radiological and histomorphometric analyses. The authors suggested that the treatment was effective as a result of the inhibition of MDA-MB-231 cell proliferation and expression of phospho-Stat3, VEGF, and RANK, although they also showed a significant decrease in the number of OC. At this point, we speculate that the observed inhibitory effects on BM formation were also due to a blockage of paracrine circuits between tumor-stimulated OB and OC in the acid microenvironment, thereby ultimately impairing OC formation. This hypothesis is in agreement with our data showing that, when added to acid-stressed OB, both TCZ and anti-IL-8 antibody treatment can revert the pro-osteolytic phenotype of OB.

We believe that our data clearly demonstrate an unexplored mechanism of tumor-induced osteolysis in BM patients and further validate the use of TCZ, or IL-6 and IL-8 inhibitors to treat BM, especially for those patients with high circulating level of these cytokines, or high FDG-PET uptake that would imply a high tumor glycolytic rate and, thus, high intratumoral acidosis (Longo et al., 2016).

## Conclusion

Altogether, our data suggest a novel model of the osteolytic vicious cycle in BM that takes into consideration the role of acidosis in the different and complex interplays between tumor cells and bone cells: the acidification of the BM microenvironment activates a feeding process for tumor expansion and bone destruction by inducing, on the one hand, the invasion of cancer cells and the attraction of osteoclastic precursors, and on the other, the secretion of pro-osteoclastogenic modulators from inflammatory OB, thereby further supporting the vicious cycle of tumor progression and osteolysis, ultimately disrupting the bone remodeling balance.

Results obtained from this study underline the complexity of the tumor microenvironment that also includes chemical–physical features that can strongly modulate cellular behavior and the progression of the disease. These should be then all taken into consideration in a more integrated and holistic approach for the development of novel therapeutic strategies for the treatment of patients with BM.

## Data Availability Statement

The original contributions presented in the study are included in the article/supplementary material, further inquiries can be directed to the corresponding author/s.

## Ethics Statement

The studies involving human participants were reviewed and approved by the Institutional Ethical Committee approval (n. 0037602 of 14.11.2013). The patients/participants provided their written informed consent to participate in this study. The animal study was reviewed and approved by National Committee for Animal Experiments (license number ESAVI-2077-04 10 07-2014).

## Author Contributions

SA, NB, and GDP conceptualized the study. GDP formulated the methodology. RG performed the formal analysis. CE, LM, TI, JT, and RG done the resource gathering (enrollment of patients and serum collection). SA and GDP performed the data curation. SA and GDP wrote and prepared the original draft. SA, GDP, RG, CE, LM, TI, JT, and NB reviewed and edited the original draft. SA performed the supervision and project administration. SA and NB acquired the funding. All authors have read and agreed to the published version of the manuscript.

## Conflict of Interest

The authors declare that the research was conducted in the absence of any commercial or financial relationships that could be construed as a potential conflict of interest.

## Footnotes

1 http://www.roche-applied-science.com

2 https://clinicaltrials.gov/

## Abbreviations

BM: bone metastasis
OB: osteoblast
OC: osteoclast
RANKL: receptor activator of nuclear factor kappa- B ligand
TGF- β: transforming growth factor-beta
VEGF: vascular endothelial growth factor
IGFs: insulin-like growth factors
PTHrP: parathyroid related protein
IL11: interleukin 11
TNF- α: tumor-necrosis factor-alpha
PGE2: prostaglandin E2
Runx2: runt-related transcription factor 2
V-ATPase: vacuolar-H^+^-ATPase
TRACP: tartrate-resistant acid phosphatase
TRAF6: TNF receptor-associated factor 6
IL-8: interleukin 8
MCP-1: monocyte chemoattractant protein-1
IL-6: interleukin 6
hOC: human osteoclasts
PPR: proton production rate
CM: conditioned medium
CA9: carbonic anhydrase 9
hOB: human osteoblasts
PBMC: peripheral blood mononuclear cells
M-CSF: macrophage colony-stimulating factor
TRACP5b: tartrate-resistant acid phosphatase 5b
CTX: cross-linked telopeptide of type I collagen
CRP: C-reactive protein
TCZ: tocilizumab neutralizing anti-IL-6 monoclonal antibody
PKM2: pyruvate kinase isoform M2P
HRE: hypoxia response element
LDHA: lactate dehydrogenase A
IL6R: IL-6 receptor
BM-MSC: human MSC from bone marrow
HMBS: hydroxymethylbilane synthase
β -actin: beta-actin.
